# Re-calibration of flow cytometry standards for plant genome size estimation

**DOI:** 10.3389/fpls.2025.1548766

**Published:** 2025-10-13

**Authors:** Abhishek Soni, Robert J. Henry

**Affiliations:** ^1^ Queensland Alliance for Agriculture and Food Innovation, The University of Queensland, St Lucia, QLD, Australia; ^2^ ARC Centre of Excellence for Plant Success in Nature and Agriculture, The University of Queensland, St Lucia, QLD, Australia

**Keywords:** flow cytometry, plant genome size, recalibration, genome assembly, haplotypes, Nipponbare rice, reference standards, consensus genome assembly

## Abstract

Flow cytometry (FCM) and genome sequencing are complementary methods for estimating plant genome size (GS). However, discrepancies between the GS estimates derived from genome assemblies and FCM create ambiguity regarding the accuracy of these approaches. Approximately 12,000 plant GS measurements have been reported, with hardly any of them based on genome assemblies. Currently, FCM is the most frequently used method. Accurate GS estimation by FCM relies on internal standards with known GS values. However, previous GS calibrations, often based on incomplete reference genome assemblies, have led to significant discrepancies in GS estimates. Historically, the GS of a diploid plant species was estimated by doubling the size of a consensus genome assembly. However, consensus assemblies collapse homologous chromosomes into a single sequence, typically favouring the larger haplotype and potentially overestimating GS, especially in highly heterozygous species. Here, we applied haplotype-resolved genome assemblies to accurately recalibrate the reference standards. We utilized a recent gapless, telomere-to-telomere (T2T) consensus and the most complete phased genome assemblies of the Nipponbare rice as a primary standard to recalibrate five commonly used plant standards. Using the consensus genome as a reference revealed an overestimation of over 30% in widely used previous GS estimates for *Pisum sativum* and *Nicotiana benthamiana*, approximately 18% for *Arabidopsis thaliana*, and 5% for *Sorghum bicolor* and *Gossypium hirsutum*. The GS estimates based on phased haplotype assemblies suggested an additional 6%–7% overestimation. Haplotype-resolved genome assemblies allow the recalibration of GS estimates with the potential to yield more accurate values by capturing haplotype-specific variations previously missed in consensus assemblies.

## 1 Introduction

Flow cytometry (FCM) is a standard approach for the estimation of plant genome size (GS) by measuring the relative fluorescence of the fluorochrome-labelled nucleus against an internal standard of known GS (Galbraith et al., 1983; Doležel et al., 2007a; Sliwinska et al., 2022; Temsch et al., 2022; Soni et al., 2025). GS is typically expressed as C-values, where the DNA content of a non-replicated haploid nucleus is referred to as the “1C-value”. This value can be measured in picograms (pg) or million base pairs (Mbp), with 1 pg being equivalent to 978 Mbp (Doležel et al., 2003). The accuracy of GS estimates is critical in genome sequencing, evolutionary studies, breeding, and biodiversity conservation for efficient allocation of sequencing resources and understanding of the adaptive significance of GS variations (Greilhuber et al., 2005; Bennett and Leitch, 2011; Pellicer et al., 2018). In addition, FCM has been used in plant taxonomy, where DNA content variation aids in species delimitation and systematics (Rosenbaumová et al., 2004; Pecinka et al., 2006; Kron et al., 2007; Suda et al., 2007). The accuracy of FCM-based GS estimation depends on selecting internal standards with precisely and accurately known GS values (Bennett and Smith, 1976; Doležel and Bartoš, 2005; Doležel and Greilhuber, 2010; Sliwinska et al., 2022; Temsch et al., 2022). Traditionally, the GS of internal standards was verified by comparing the relative fluorescence of various standards, often including reference genomes of non-plant organisms such as humans and chickens (Van'T Hof, 1965; Bennett and Smith, 1976, 1991; Doležel et al., 1998; Praça-Fontes et al., 2011). The calibration of a plant standard against non-plant genomes may not be reliable due to differential staining properties (Doležel and Greilhuber, 2010; Sliwinska et al., 2022), suggesting the use of plants as primary standards to calibrate the GS of plant reference standards.

An accurate estimation of the GS of primary standards requires the achievement of complete genome assembly (Doležel and Greilhuber, 2010). Ideally, a complete genome assembly refers to an assembly where the entire genome is fully represented, spanning from telomere to telomere (T2T), with minimal gaps or unplaced sequences. It should be haplotype-resolved, accurately phasing both alleles, and capturing structural variations. This level of completeness has only recently become achievable with the advent of long-read sequencing (Murigneux et al., 2020; Nurk et al., 2022; Chen et al., 2023; Gladman et al., 2023; Shang et al., 2023; Mo et al., 2024). Moreover, the integration of the Hi-C and long sequencing reads now allows for the generation of fully phased assemblies, enabling a more complete representation of the genome structure of diploid and polyploid species. Phased genome assemblies involve the reconstruction of two distinct haplotypes, providing a more accurate representation of genetic variation compared to consensus assemblies, which merge the two haplotypes into a single sequence (Guk et al., 2022). Recent studies have identified significant structural variations in haplotypes due to the presence of insertions, deletions, and chromosomal rearrangements that are specific to individual haplotypes (Koren et al., 2018; Guk et al., 2022; Abdullah et al., 2024; Li et al., 2024; Wilkinson et al., 2025). Consideration of these variations could contribute to more accurate genome size estimation. However, previous recalibrations of the plant standards using the Nipponbare rice overlooked these variations (Veselý et al., 2013; Šmarda et al., 2014; Temsch et al., 2022; Skaptsov et al., 2024). Consequently, these estimates were not only based on incomplete assembly but also failed to account for haplotype variations, leading to the misrepresentation of the actual GS of the rice (Veselý et al., 2013; Šmarda et al., 2014; Temsch et al., 2022; Skaptsov et al., 2024). Abdullah et al. (2024) found a significant difference of 31 Mbp in the two haplotypes of the Nipponbare rice. The total size of the two haplotypes of the Nipponbare rice was (2C = 0.743 pg), which was 7% smaller than the previously used GS value of the Nipponbare rice (Veselý et al., 2013; Šmarda et al., 2014; Temsch et al., 2022; Skaptsov et al., 2024) and 5.7% smaller than that of the most complete consensus genome assembly (Shang et al., 2023). In addition, the currently available complete consensus genome assembly was 3.4% larger than the earlier short-read assembly (Sasaki, 2005; Shang et al., 2023). These differences are particularly significant for species with smaller genomes, such as *Arabidopsis thaliana*.

Here, we used the most complete consensus assembly (2C = 0.788 pg) and the total of the two haplotypes (2C = 0.743 pg) of the Nipponbare rice as a primary reference standard (Shang et al., 2023; Abdullah et al., 2024) to recalibrate five plant standards with ~28-fold diversity of GS with 2C values ranging from 0.272 to 7.65 pg.

## 2 Methods

### 2.1 Selection of species

Young seedlings of *Oryza sativa* ssp. *japonica* cv. Nipponbare, *Sorghum bicolor* cv. BTx623, *Pisum sativum* cv. Torstag, *A. thaliana* Col-0, *Nicotiana benthamiana* LAB, and *Gossypium hirsutum* cv. Siokra were cultivated under controlled conditions. These species were chosen because of their easy accessibility, availability of previously reported GS estimates generated by different techniques, and high-quality genome assemblies based on long-read sequencing technologies.

### 2.2 Genome size estimation of Nipponbare rice

The genome size of the Nipponbare rice was determined using three genome assemblies: (α) the previously available consensus assembly (2C = 0.795 pg) (Sasaki, 2005), (β) the complete consensus assembly (2C = 0.788 pg) (Shang et al., 2023), and (γ) the haplotype-resolved assembly (2C = 0.743 pg) (Abdullah et al., 2024). The earlier consensus assembly, although widely used for estimating the GS of diploid rice, was incomplete. The more recent gapless, T2T assemblies and haplotype-resolved assemblies address these gaps, providing more accurate estimates by incorporating haplotype variations. The rice genome, assembled using a hybrid approach of HiFi reads for high accuracy and ultra-long Oxford Nanopore Technology (ONT) reads for resolving complex, repetitive regions, achieved a consensus accuracy of approximately one error per 5 million bases (Q63) (Zhang et al., 2022; Sahu and Liu, 2023; Shang et al., 2023; Xie et al., 2024). Similarly, hybrid sequencing approaches have been employed to assemble other gapless *Oryza* genomes (Song et al., 2021; Zhang et al., 2022).

### 2.3 Flow cytometry

A one-step protocol was used to isolate intact nuclei from fresh plant material (Galbraith et al., 1983; Doležel et al., 2007a; Soni et al., 2025). Approximately 40 mg of both the internal standard and test species were co-chopped in 800 µL of ice-cold modified buffer (Mb01), which was prepared as described by Sadhu et al. (2016). The nuclei suspension was filtered through a 40-µm nylon cell filter, and 400 µL of the suspension was transferred to a 5-mL round-bottom Fluorescence-Activated Cell Sorting (FACS) tube. A staining buffer consisting of 20 µL propidium iodide (1 mg/mL) (~50 ppm final concentration) and 0.2 µL RNase (1 mg/mL) was added to the suspension, and the tube was kept on ice until fluorescence measurement. Propidium iodide-labelled nuclei were excited using a 488-nm blue laser, and fluorescence was recorded using a Becton Dickinson LSRFortessa X-20 Cell Analyzer equipped with a 695/50 nm bandpass filter (Koutecký et al., 2023). Data were collected on a linear scale with a trigger threshold set at 5,000, recording at least 600 events per peak and a total of 2,000 events at a flow rate of 12 µL/min, yielding 10–20 events per second. Signal amplification was achieved by setting the Forward Scatter (FSC) detector voltage to 320, the Side Scatter (SSC) voltage to 179, and the fluorescence detector voltage to 405. FSC detector voltage to 320, the SSC voltage to 179, and the fluorescence detector voltage to 405. For *Arabidopsis*, the fluorescence detector voltage was set to 495. Forward scatter and side scatter were recorded on a logarithmic scale to identify fluorescence peaks, and pulse width vs. pulse height plots were used to eliminate aggregates and debris. The linear scale acquisition of fluorescence preserves the proportionality of fluorescence intensity and allows accurate coefficient of variation (CV) calculations (Koutecký et al., 2023). The acquisition of 2,000 events per sample aligns with established guidelines (Koutecký et al., 2023). Recent evaluations have shown that such counts are sufficient for high-quality histograms, providing accurate and precise results without loss of precision (Koutecký et al., 2023). Consistent manual gating was applied to remove debris across replicates, with a focus on selecting the minimum required number of events from the middle of the population distribution and a CV of less than 5% (Loureiro et al., 2007). Manual gating remains a widely accepted standard in plant genome size estimation, although automated algorithms are increasingly being adopted for plant FCM datasets to reduce operator bias (Smith et al., 2018). In this study, the high resolution of fluorescence peaks supported the use of manual gating, which minimized potential errors. Applying the same gating strategy across all replicates and species ensured the reproducibility and consistency of the results. To ensure the accuracy of recalibration, three biological replicates per species were performed to calculate the average genome size. Genome size was calculated using the following equation:

Genome size (2C/pg) = (Mean fluorescence of sample species/Mean fluorescence of internal standard) × 2C (pg) value of internal standard.

Subsequently, the recalibrated estimates were cross-validated against the genome assembly data, previous estimates, and recalculated estimates using an updated human genome size.

### 2.4 Experimental design

A direct comparison between pea and rice, which differ in GS by approximately 12-fold, would likely have caused non-linearity in the flow cytometric fluorescence signal, thereby compromising the accuracy of GS estimations. To maintain linearity and avoid poor peak resolution, the ratio of fluorescence intensities between sample and standard should remain within approximately threefold (Doležel et al., 1998, 2007; Sliwinska et al., 2022). To address this, a cascade approach was employed to ensure that fluorescence effects remained within an acceptable range. In this approach, rice was used as the primary standard for calibrating *S. bicolor* and *A. thaliana.* The recalibrated sorghum reference was then employed to calibrate *G. hirsutum*, which in turn was used to calibrate *P. sativum.* Finally, the recalibrated pea standard was used for calibrating *N. benthamiana*, ensuring more reliable and accurate GS determinations.

To contextualize and compare GS estimates across different studies, a comprehensive literature review was conducted. Published GS values derived using various methods, including FCM, genome sequencing, K-mer analysis, spectrophotometry, and Feulgen microdensitometry, were extracted from literature and a C-value database (https://cvalues.science.kew.org/) (Pellicer and Leitch, 2020). The NCBI database (https://www.ncbi.nlm.nih.gov/home/genomes/) was searched for the most complete genome assemblies of the selected species. Where applicable, the GS values, which had been reported in varying units, such as mega base pairs (Mbp), or as 1C or 3C DNA content, were standardized by converting them to 2C values expressed in picograms (pg) using the established conversion factor of 1 pg = 978 Mbp (Doležel et al., 2003). Subsequently, the diversity in these estimates was visualized using R (Ihaka and Gentleman, 1996; Wickham, 2011).

Since non-plant genomes, particularly those derived from human male leukocytes, have historically been used to recalibrate plant standards (Tiersch et al., 1989; Doležel et al., 1992, 1998), first, historical GS estimates were examined for the human male genome. The updated human GS (2C = 6.15 pg) used for recalibration in this study was based on the most complete, T2T human genome assemblies currently available. A detailed justification and comparison of several high-quality human genome assemblies available through the NCBI database, including T2T-CHM13 v2.0, HG002 Ref.pat, GRCh38.p14, and Han1, is provided in **Note S1**. The quality of the genome assemblies and the sequencing technologies were assessed through a literature review. The T2T-CHM13 v2.0 assembly, with 1C = 3.075 pg (3.075 Gb), was selected due to its completeness and coverage metrics (Nurk et al., 2022; Rhie et al., 2023) and was cross-validated against additional diploid and haploid assemblies (Jarvis et al., 2022; Chao et al., 2023).

Several plant standards were historically calibrated using chicken erythrocytes (Galbraith et al., 1983; Arumuganathan and Earle, 1991; Johnston et al., 1999), and the chicken GS values were originally derived using human leukocytes (Tiersch et al., 1989). Arumuganathan and Earle (1991) calibrated plant standards based on a chicken genome size of 2C = 2.33 pg, which was previously estimated by Galbraith et al. (1983) using calf thymus DNA as the reference. Subsequently, Johnston et al. (1999) evaluated the genome size of chickens using three different tissue sources and reported 2C values ranging from 2.48 to 3.01 pg. Given this variability and the historical interdependence between humans and chickens, we recalculated the chicken GS using the human/chicken fluorescence ratio reported by Tiersch et al. (1989), in combination with the updated human GS (2C = 6.15 pg). This resulted in a revised estimate of 2C = 2.20 pg for chickens. The recalculated GS values of plant standards from both Arumuganathan and Earle (1991) and Johnston et al. (1999), using historical fluorescence ratio and updated chicken GS, were used as reference points to guide our own FCM measurements for comparison with historical GS estimates.

Finally, the recalibrated GS estimates (α) based on old rice assembly, (β) based on recent gapless rice assembly, and (γ) based on haplotype-resolved rice assembly were compared with previous GS estimates, recalculated GS estimates, and the GS estimates from the most complete genome assemblies using linear regression analysis. In each comparison, the recalibrated genome size (γ) served as the independent variable, while other estimates were the dependent variable. The model fit was evaluated using the coefficient of determination (R^2^) and residual analysis. All analyses were performed in R (v 4.2.2) using the lm() function. Scatter plots with regression lines were generated using the ggplot2 package (v 3.5.1).

## 3 Results

### 3.1 Recalibrated GS estimates

Recalibrated estimates based on the previous rice genome (α) and the recent consensus genome (β) differed by approximately 1% across all the species (**Table 1**). However, the recalibrated estimates based on haplotype-resolved rice assembly (γ) were 6%–7% smaller than the α and β estimates of the species. Recalibrated values (γ) for *A. thaliana*, 0.272 ± 0.002 pg, were approximately 9% smaller than the previous estimate (2C = 0.297 pg) based on chicken erythrocytes (2C = 2.33 pg), whereas they were 18% smaller than the value 2C = 0.321 pg calibrated against *Caenorhabditis elegans* (2C = 0.204 pg) (**Table 1**). The recent consensus genome assemblies with 2C = 0.291 and 0.286 pg supported the precision and accuracy of the consensus assembly based on recalibrated values (α and β) but overestimated by up to 7% relative to the haplotype-based estimation (γ) (**Table 1**). Similarly, the recalibrated GS (γ) of sorghum (2C = 1.458 pg) was nearly 2% larger than the previous, recalculated, and T2T genome assembly-based estimates (**Table 1**). Based on the recalibrated sorghum (γ), cotton was calibrated at 4.46 pg/2C, which was approximately 8% smaller than the previous GS estimate based on chicken erythrocytes (2C = 2.33 pg). The gamma-based estimate was also 6.5% lower than the value derived from the consensus genome assembly (2C = 4.75 pg) and approximately 7% lower than both the α- and β-based estimates. The recalculated value (2C = 4.59 pg) based on the updated chicken GS was 3% overvalued from the recalibrated size (γ). Using recalibrated cotton (γ), the 2C value of pea was estimated to be 7.22 pg. In contrast, the previous calibration values (2C = 9.49 pg, 9.09 pg) were approximately 31% and 26% overvalued relative to the recalibrated estimate (γ). The recalculated estimate (2C = 7.99 pg) was nearly 11% higher than the recalibrated value (γ). In addition, genome assemblies for two other cultivars of pea (2C = 7.67, 7.61 pg) corresponded well with the recalibrated values (α and β); however, they showed an approximately 6% overestimation relative to the recalibrated estimate (γ). Using an updated pea GS (γ), the recalibrated 2C estimate of *N. benthamiana* was 5.5 pg, approximately 16% smaller than the previous Feulgen densitometry-based estimate (2C = 6.4 pg), which was calibrated against onion (2C = 33.5 pg), although the recalibrated value (γ) was nearly 6% larger than the estimate based on a recent T2T consensus genome assembly of *N. benthamiana* (2C = 5.826 pg) (**Table 1**).

**Table 1:** The recalibrated GS estimates, previous estimates, recalculated estimates, and corresponding genome assembly sizes.

Species	Internal standard	Fluorescence ratio	Recalibrated 2C estimate ^α^ (pg ± SE)	Recalibrated 2C estimate ^β^ (pg ± SE)	Recalibrated 2C estimate ^γ^ (pg ± SE)	Previous 2C estimate (pg)	Recalculated 2C estimate (pg) (based on updated reference GS)	2C estimate based on consensus assembly size (2C/pg)
*Arabidopsis thaliana* (Col-0)	*Oryza sativa* ssp. *japonica* cv. Nipponbare	0.360^1^	0.291 ± 0.003	0.288 ± 0.002	0.272 ± 0.002	0.297^a1^, 0.321^a2^	0.28^c^	0.291^4^, 0.286^5^
		0.365^2^						
		0.372^3^						
*Sorghum bicolor* cv. BTx623	*O. sativa* ssp. *japonica* cv. Nipponbare	1.958^1^	1.56 ± 0.005	1.545 ± 0.004	1.458 ± 0.004	1.48^a1^	1.49^c^	1.339^6a^, 1.279^6b^ 1.481^6c^, 1.486^6c^
		1.952^2^						
		1.972^3^						
*Gossypium hirsutum* cv. Siokra	*Sorghum bicolor* cv. BTx623	3.11^1^	4.78 ± 0.04	4.733 ± 0.04	4.46 ± 0.04	4.8^a1^ 4.3 ^a2^	4.59^c^	4.75^7^
		3.02^2^						
		3.06^3^						
*Nicotiana benthamiana* LAB	*Pisum sativum* cv. *Torstag*	0.767^1^	5.89 ± 0.02	5.828 ± 0.02	5.5 ± 0.02	6.4^d^	5.83^e^	5.826^8^
		0.759^2^						
		0.759^3^						
*Pisum sativum* cv. Torstag	*G. hirsutum* cv. Siokra	1.63^1^	7.73 ± 0.03	7.650 ± 0.03	7.22 ± 0.03	9.09^b1^, 9.49^b2^	7.99^d^	7.67^9^, 7.61^10^
		1.61^2^						
		1.61^3^						

GS, genome size.

^α^ Based on the previous consensus rice genome assembly (2C = 0.795 pg) (Sasaki, 2005).

^β^ Based on the complete consensus rice genome assembly (2C = 0.788 pg) (Shang et al., 2023).

^γ^ Based on the total of two haplotypes (2C = 0.743 pg) (Abdullah et al., 2024); a1 = calibrated against chicken genome (2C = 2.33 pg); a2 = calibrated against *Caenorhabditis elegans* (2C = 0.204 pg) (Bennett et al., 2003); b1 = calibrated against previous human male genome (2C = 7 pg); b2 = as per Jordan et al. (2015); c = based on the current chicken genome (2C = 2.20 pg) derived from human/chicken ratio from Tiersch et al. (1989) and updated human male GS; d = based on the current human male genome (2C = 6.15 pg); e = based on the previous estimate of onion (2C = 30.54 pg) derived from onion/human fluorescence ratio from Doležel et al. (1992) and updated human male GS (2C = 6.15 pg); 1, 2, 3 = replicate numbers; 4 = Lian et al. (2024); 5 = NCBI (2024); 6a = Paterson et al. (2009); 6b = Mccormick et al. (2018); 6c = Bao et al. (2024); 7 = Chang et al. (2024); 8 = Chen et al. (2024); 9 = Kreplak et al. (2019); 10 = Yang et al. (2022).

**Figure 1:**
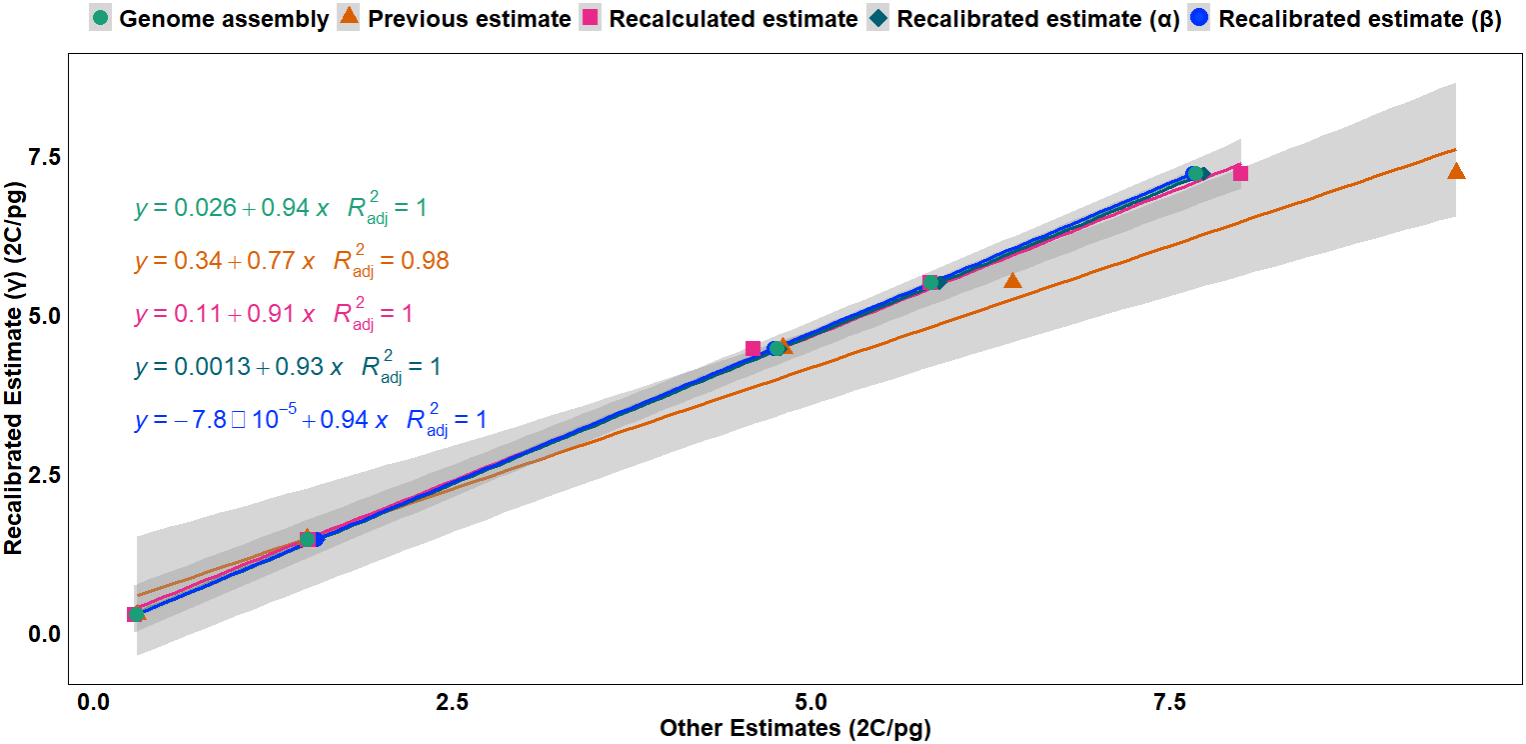
Regression analysis of the recalibrated GS estimates (γ) based on the haplotype-resolved genome assembly (2C = 0.743 pg) against multiple calibration approaches: genome assembly-based estimates, previous estimates, recalculated estimates, recalibrated estimate (α) based on previous genome assembly (2C = 0.795 pg) (Sasaki, 2005), and recalibrated estimate (β) based on the most complete consensus genome assembly (2C = 0.788 pg). Higher slope values and smaller intercepts indicate stronger linear relationships between the recalibrated estimate, recalculated estimates, and genome assembly size. The lines represent linear regressions with corresponding equations and adjusted R^2^ values. GS, genome size.

Regression analysis (**Figure 1**) revealed a strong linear relationship between the recalibrated estimates (γ) based on haplotype assembly of rice (2C = 0.743 pg) and the other calibration methods, with adjusted R^2^ values ranging from 0.98 to 1, indicating consistency between recalibrated estimates (α, β, and γ), genome assembly sizes, and the recalculated estimates based on the updated human and chicken GS values, suggesting near-equivalence between these methods except for the overestimation from the previous estimates based on outdated reference genome sizes. Furthermore, the slopes indicated that GS estimates from other methods were slightly overestimated when compared to the haplotype-based recalibrated estimates (γ) (**Figure 1**, **Supplementary Figure S1**).

### 3.2 Discrepancies in the previous GS estimates

**Figure 2:**
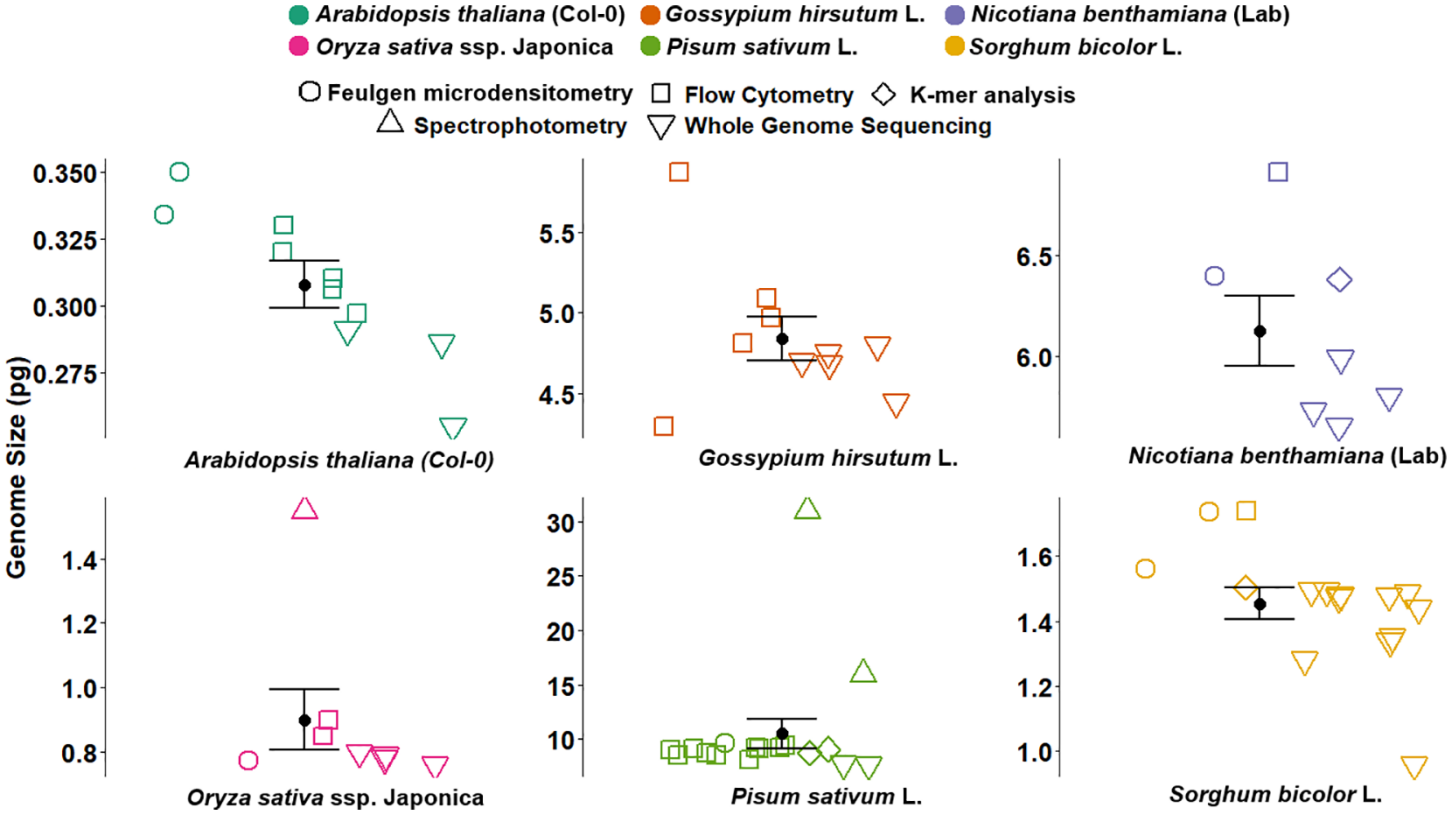
Genome size estimates for six plant reference species obtained using various methods, including Feulgen microdensitometry (open circles), flow cytometry (squares), spectrophotometry (triangles), K-mer analysis (diamonds), and whole genome sequencing (inverted triangles). Coloured points represent previously published GS estimates compiled from the literature, with the corresponding references, method, and values detailed in **Supplementary Table S1**. Each subplot represents a different species: *Arabidopsis thaliana* (Col-0), *Oryza sativa* ssp. *japonica*, *Gossypium hirsutum* L., *Pisum sativum* L., *Nicotiana benthamiana* (LAB), and *Sorghum bicolor* L. Black dots with error bars indicate the average GS estimates with standard error. The figure illustrates variation in GS estimates across methods and highlights the consistency and precision of the historical values. GS, genome size.

Previously, methods like Feulgen microdensitometry, spectrophotometry, and cell cycle analysis contributed to the foundational work in GS estimation and the calibration of reference standards (Van'T Hof, 1965; Bennett and Smith, 1976). Feulgen microdensitometry involved staining the DNA and quantifying the dye bound to the nuclei, while spectrophotometry measured light absorbance to estimate the amount of DNA, and cell cycle analysis inferred GS based on the timing of replication and division phases. However, the accuracy of these methods relied heavily on the correct GS of the reference standard. For instance, Van'T Hof (1965) estimated the GS for onion (2C = 33.5 pg) based on the length of the cell cycle and the proportionality of the frequency of 2C, intermediate, and 4C cells to the respective duration of the cell cycle stages. However, this method was later found to be less accurate in the estimation of absolute GS values (Bennett and Smith, 1976). Bennett and Smith (1976) used onion (2C = 33.5 pg) to calibrate eight angiosperm species to be nominated as internal standards, including pea (2C = 9.72 pg). Later, the GS of pea varied threefold in several studies employing different GS estimation techniques (**Figure 2**) (Lyndon, 1963; Bennett and Smith, 1976, 1991; Michaelson et al., 1991; Doležel et al., 1992; Baranyi et al., 1996; Doležel et al., 1998; Johnston et al., 1999; Doležel and Greilhuber, 2010; Kreplak et al., 2019; Yang et al., 2022). However, due to the lack of quality plant genomes, these values could not be verified for accuracy. Although given their precision from different studies, the values were often used to estimate the GS of plant species. The calibration of pea against human male leukocytes (2C = 7 pg) as reported by Tiersch et al. (1989) demonstrated high precision, leading to the establishment of pea (2C = 9.09 pg) as a reference standard (Doležel et al., 1992, 1998). This value has since been widely applied across a variety of plant species for GS estimation and as a guide for genome sequencing studies of pea (Kreplak et al., 2019; Yang et al., 2022).

Similarly, significant discrepancies were observed in the calibrated GS of the common plant standards (**Figure 2**, **Supplementary Table S1**). The GS of *A. thaliana* in previous studies varied from 0.255 to 0.334 pg (Bennett and Smith, 1991; Galbraith et al., 1991; Krisai and Greilhuber, 1997; The Arabidopsis Genome, 2000; Bennett et al., 2003; Hou et al., 2022; Kang et al., 2023; Lian et al., 2024; NCBI, 2024), allotetraploid cotton varied from 4.29 to 5.09 pg (Arumuganathan and Earle, 1991; Hendrix and Stewart, 2005; Li et al., 2015; Hu et al., 2019; Wang et al., 2019; Huang et al., 2020; Chang et al., 2024), *N. benthamiana* LAB varied from 5.64 to 6.92 pg (Narayan, 1987; Hussain et al., 2021; Kurotani et al., 2023; Ranawaka et al., 2023; Chen et al., 2024; Ko et al., 2024; Wang et al., 2024), sorghum varied from 0.975 to 1.735 pg (Laurie and Bennett, 1985; Johnston et al., 1999; Paterson et al., 2009; Deschamps et al., 2018; Mccormick et al., 2018; Tao et al., 2021; Wang et al., 2021; Bao et al., 2024; Ding et al., 2024), and *O. sativa* ssp. *japonica* cv. Nipponbare varied from 0.77 to 1.55 pg (Bennett and Smith, 1976; Iyengar and Sen, 1978; Arumuganathan and Earle, 1991; Bennett and Smith, 1991; Goff et al., 2002; Sasaki, 2005; Shang et al., 2023; Abdullah et al., 2024) (**Figure 2**). Based on the recalibrated GS estimates, the discrepancies in past GS estimates can be attributed to the inaccurate calibration of reference standards, such as the outdated human male genome size of 2C = 7 pg or the chicken genome size of 2C = 2.33 pg, as well as the lack of verification against plant genomes, incorrect GS determination from incomplete genome assembly, and ignoring variations between haplotypes.

### 3.3 Source of discrepancies in GS estimates

Tiersch et al. (1989) calibrated several reference standards against the human male genome (2C = 7 pg), including the chicken genome (2C = 2.33 pg). Both human and chicken genomes were extensively used as primary standards for verifying the GS for plant internal standards (Bachmann, 1972; Galbraith et al., 1983; Tiersch et al., 1989; Doležel et al., 1998; Doležel and Greilhuber, 2010) (**Table 2**). However, after 20 years of advancements, the human male genome is now fully sequenced and considered complete and gapless (2C = 6.15 pg) (**Note S1**) (Miga et al., 2020; Jarvis et al., 2022; Nurk et al., 2022; Chao et al., 2023; Rhie et al., 2023). A comprehensive analysis revealed that the T2T-CHM13 assembly maintains uniform coverage across the genome, with 99.86% of the sequence within three standard deviations of the mean coverage for HiFi and ONT reads (Nurk et al., 2022). Excluding the ribosomal DNA (rDNA) sequences, this uniformity further improves the coverage to 99.99%. Despite this high level of completeness, some regions of the genome remain associated with potential issues due to low coverage, low confidence, or known heterozygous sites due to the limitations of both ONT and HiFi read challenges in dealing with telomeric regions and Guanine-Cytosine (GC)-rich regions, respectively (Nurk et al., 2022; Li and Durbin, 2024). These potential issues encompass only 0.3% of the total assembly length, equivalent to approximately 9.165 Mbp. The complete genome revealed an overestimation of 14% in the previous GS estimates of the male genome (**Note S1**). Therefore, based on the chicken/human ratio of 0.357 from Tiersch et al. (1989), the chicken GS was recalculated to be 2C = 2.20 pg. This implied that previous plant standard calibrations using the chicken genome (2.33 pg) were overestimated by 6% (Galbraith et al., 1983; Tiersch et al., 1989; Arumuganathan and Earle, 1991; Doležel and Greilhuber, 2010). **Table 2** presents the recalculated estimates for some plant standards based on updated human and chicken genome sizes. Estimates for sorghum, cotton, and pea were in accordance with the recalibrated estimates. However, a higher calibration value of 2C = 7.99 pg for ‘Ctirad’ pea could be cultivar-specific. Veselý et al. (2011) calibrated the ‘Citrad’ pea (2C = 8.02 pg) against the Nipponbare rice (2C = 0.795 pg). This was recalculated to 7.48 pg using the haplotype-resolved rice genome (2C = 0.743 pg), still giving a 7% overestimation, implying that the differential staining properties of plant and non-plant genomes could also add to inaccuracies. In addition, other factors such as the presence of secondary metabolites (e.g., phenolics and tannins) and technical aspects, including fluorochrome selection, buffer composition, instrumentation, and methodological differences, may also affect genome size estimates if not properly controlled (Greilhuber, 1986; Doležel et al., 1992, 1998; Price et al., 2000; Loureiro et al., 2006a; b; 2007).

**Table 2:** Previous and recalculated DNA contents of the plant reference standards calibrated against old and current genome size estimates of human male leukocytes and chicken erythrocytes.

Species	Previous 2C DNA content (pg)	Florescence ratio	Recalculated 2C DNA content (pg)
*Raphanus sativus* cv. Saxa	1.11^a^	0.16	0.98^c^
*Solanum lycopersicum* cv. ‘Stupike’	1.96^a^	1.77	1.72^c^
*Sorghum bicolor* L.	1.57–1.62^b^	0.67–0.70	1.47–1.54^d^
*Solanum lycopersicum* cv. Stupické Polní Rané’ *Glycine max* L.	2.50^a^	1.28	2.20^c^
*Gossypium hirsutum*	4.45–4.98^b^	1.91–2.14	4.2–4.71^d^
*Zea mays* cv. Polanka	5.43^a^	2.17	4.77^c^
*Z. mays* L.	4.75^b^	2.04	4.49^d^
*Pisum sativum* cv. Ctirad	9.09^a^	1.67	7.99^c^
*P. sativum* L.	8.07^b^	3.46	7.61^d^
*Secale cereale* cv. Dankovske	16.19^a^	2.31	14.22^c^
*Vicia faba* cv. Inovec	26.90^a^	3.84	23.63^c^
*Allium cepa* cv. Alice	34.76^a^	4.965	30.53^c^

a Calibrated against human male leukocytes (2C = 7 pg) (Tiersch et al., 1989; Doležel et al., 1998).

b Calibrated against chicken erythrocytes (2C = 2.33 pg)* (Galbraith et al., 1983).

c Recalculated estimate using updated human male genome (2C = 6.15 pg) (Nurk et al., 2022).

d Recalculated estimate using updated chicken genome (2C = 2.2 pg).

* Values were normalized as per 1 pg = 978 Mbp.

## 4 Discussion

The 2005 assembly of the Nipponbare rice has been used to determine the GS of the rice (2C = 0.795 pg) for FCM (Sasaki, 2005; Veselý et al., 2011, 2013; Šmarda et al., 2014; Temsch et al., 2022; Skaptsov et al., 2024). However, this assembly was based on 12 scaffolds of 370-Mb length, while the remaining 18.8 Mb was estimated as gaps (Sasaki, 2005). With the use of long-read sequencing, the recently assembled consensus genome, consisting of 12 complete gapless, T2T chromosomes with a total length of 385.7 Mb, was nearly 0.9% smaller than the previous assembly. The recent genome was assembled using a hybrid assembly strategy including PacBio HiFi, ultra-long ONT, and Hi-C reads (Shang et al., 2023). Abdullah et al. (2024) used the HiFi and Hi-C reads to generate a haplotype-resolved assembly. High mapping rates >99.6% of raw reads to the assembled genome, the presence of 99.88% of the Benchmarking Universal Single-Copy Orthologs (BUSCO) gene set, and T2T chromosomes represented the completeness of the Nipponbare rice genome (Shang et al., 2023).

The previous consensus assembly-based estimate for rice was approximately 7% overestimated from the average of the two haplotypes since collapsed assembly provided a single representation of the chromosomal structures without considering the chromosomal arrangement or structural variations (Chin et al., 2016; Koren et al., 2018; Zhang et al., 2019; Guk et al., 2022; Li et al., 2024). Therefore, a haplotype-resolved assembly is likely to be a more realistic representation of the GS for a diploid or polyploid species (Guk et al., 2022). The integration of Hi-C data with HiFi reads supports the highly accurate phasing of assemblies (Guk et al., 2022; Nakandala et al., 2023; Li et al., 2024). Using similar technologies, Haplotype 1 (379.2 Mb) and Haplotype 2 (348 Mb) were assembled with high contiguity (N50 = ~30 Mb) (Abdullah et al., 2024). All chromosome-level scaffolds in Haplotype 1 contained telomeres on both ends except chromosome 9. In contrast, chromosomes 8, 9, and 11 in Haplotype 2 had one telomere, and these scaffolds had chromosome-level lengths of 26.2, 21.1, and 29.3 Mbp, respectively. However, these chromosomes were 28.6 and 30.7 Mbp in the complete consensus genome, thus accounting for a difference of less than 2%. Guk et al. (2022) reviewed several haplotype-resolved assemblies reporting differences in the assembly size of the haplotypes, and the differences were largely due to structural variations, which have been associated with phenotypic variations in many other plants like potato, apple, and tea (Zhang et al., 2019; Zhou et al., 2020; Zhang et al., 2021). In the absence of a haplotype-resolved genome, the actual genome size of the species cannot be determined. The collapsed genome may exceed the length of both haplotypes (Wijesundara et al., 2024). Structural variations between haplotypes (Wilkinson et al., 2025) are likely to be especially important in heterozygous species with large numbers of structural differences between the haplotypes.

The recalibrated GS estimates in this study revealed that earlier estimates, particularly those derived from flow cytometry and consensus genome assemblies, were overestimated. For all five species, the consensus genome assemblies were larger than the gamma-based recalibrated estimates. This overestimation likely stems from the way consensus assemblies collapse homologous chromosomes by prioritizing the longer haplotype while disregarding shorter allelic variants. As a result, the final consensus genome reflects an artificially inflated chromosome size compared to the actual haploid structure. Recalibrated estimates were cross-validated against the estimates generated from different studies. For example, the recalculated GS estimate of *A. thaliana* (2C = 0.28 pg) from Arumuganathan and Earle (1991) closely matched the recalibrated GS (β) based on the complete consensus rice assembly (β). Similarly, a recalculated estimate of 0.286 pg from a lamp-based FCM from Doležel et al. (1998) aligned closely with the recalibrated value (β). However, α- and β-based values were nearly 6% overestimated relative to the γ-based value. Previous GS values for *A. thaliana* (2C = 0.306 pg) against *Drosophila melanogaster* and 2C = 0.32 pg against *C. elegans* were overestimated by 12% and 18%, respectively, from the γ-based value (Bennett et al., 2003; Temsch et al., 2022). The recent complete T2T genome assembly Col-CC (GCA_028009825.2) (NCBI, 2024; Reiser et al., 2024) showed 2C values of 0.291 pg and 2C/0.286 pg. Lian et al. (2024) further validated the recalibrated α and β values since the consensus genome assemblies were 6%–7% overestimated compared to the γ-based value. This may be linked with the haplotype-based variations in the *Arabidopsis* genome. Krisai and Greilhuber (1997) calibrated the size of the *A. thaliana* genome (2C = 0.334 pg) against pea (2C = 8.84 pg) using Feulgen photometry; however, considering the recalibrated GS of pea from this study (2C = 7.22 pg), the GS of *A. thaliana* would be 0.272 pg/2C, consistent with our gamma-based estimate.

Furthermore, the GS of sorghum varied from 1.28 to 1.56 pg historically (Laurie and Bennett, 1985; Johnston et al., 1999; Paterson et al., 2009; Deschamps et al., 2018; Mccormick et al., 2018; Tao et al., 2021; Wang et al., 2021; Bao et al., 2024; Ding et al., 2024). The recalibrated GS value (γ) (2C = 1.458 pg) differed approximately 9% and 14% from earlier genome assemblies of the BTx623 cultivar, which reported values of 2C = 1.339 pg and 2C = 1.279 pg, respectively, based on short-read and Sanger sequencing (Paterson et al., 2009; Mccormick et al., 2018). These discrepancies likely reflect the limitations of short-read sequencing technologies in resolving repetitive and complex regions, resulting in incomplete assemblies and the underestimation of genome size. In contrast, recent T2T assemblies of different *Sorghum* cultivars, generated using long-read sequencing technologies, reported GS of 2C = 1.48 pg, aligning with the γ-based recalibrated values (Bao et al., 2024; Ding et al., 2024).

The GS of allotetraploid cotton in this study was recalibrated at 4.46 pg against sorghum (2C = 1.458 pg). This value was approximately 11% and 32% smaller than the previous calibration against *O. sativa* IR36 (2C = 1.01 pg) and *Hordeum vulgare* cv. Sultan (2C = 11.12 pg), respectively (Hendrix and Stewart, 2005). However, recent consensus genome assemblies using long-read sequencing technologies citing 2C = 4.75 pg from Chang et al. (2024), 2C = 4.68 pg from Huang et al. (2020), 2C = 4.80 pg from Wang et al. (2019), and 2C = 4.694 pg from Hu et al. (2019) differed by 5%–7% from the recalibrated estimate (γ). The recalibrated estimate (β), (2C = 4.73 pg), agreed with the consensus genome assemblies of cotton, further supporting the preference of the haplotype-resolved assembly for GS estimation.

The recalibrated GS for pea was 7.22 pg/2C, which was approximately 31% smaller than the earlier reported value of 9.49 pg for the same cultivar used in the GS estimation of the Proteaceae family (Jordan et al., 2015). Using the updated human male GS (6.15 pg), the recalculated GS value for ‘Ctirad’ pea (Doležel et al., 1998) was 7.99 pg, which showed a nearly 14% overestimation in the previous calibration value of pea. This result was in accordance with 2C = 8.01 pg against the previous consensus genome of the Nipponbare rice (2C = 0.795 pg) (Veselý et al., 2013; Šmarda et al., 2014; Temsch et al., 2022). However, the recent consensus genome assemblies of pea (2C = 7.61 and 7.67 pg) reflected a 6% overestimation when compared with the recalibrated γ value.

Previous FCM-based and consensus genome assembly-based GS estimates for *N. benthamiana* were approximately 16% and 6% overestimated than the recalibrated value (γ) of *N. benthamiana* (2C = 5.5 pg), respectively, although the recalibration value (β) based on the complete consensus genome of the Nipponbare rice was equal to the complete consensus genome assembly of *N. benthamiana* (Chen et al., 2024). In addition, genomes assembled using long-read technologies, citing 5.648 pg from Ko et al. (2024), 5.988 pg from Wang et al. (2024), 5.797 pg from Ranawaka et al. (2023), and 5.724 pg from Kurotani et al. (2023), were 4%–8% overestimated relative to the recalibrated γ estimate.

The accuracy of the recalibrated estimates (γ) was verified by the previous results from different laboratories and methodologies. For instance, the GS of *onion* was estimated as 35.76 pg/2C and 37.13 pg/2C against ‘Ctirad’ *pea* (2C = 9.09 pg) in two laboratories using lamp-based FCM (Doležel et al., 1998). Veselý et al. (2011) calibrated ‘Ctirad’ pea against an old Nipponbare rice assembly (α, 2C = 0.795 pg) at 8.02 pg. However, based on haplotype-resolved rice assembly (β), the recalculated value for pea was 7.48 pg. Using the recalculated ‘Ctirad’ pea, the GS for onion was recalculated as 29.42 and 30.55 pg (Doležel et al., 1998). These values were 10% smaller than the GS estimated by Van'T Hof (1965), but in accordance with the speculations of 33.5 ± 10 pg conducted using Feulgen densitometry by Bennett and Smith (1976). Moreover, an FCM-based study (Doležel et al., 1992) estimated 2C = 34.76 pg for onion using the human male genome (previously 2C = 7 pg). However, when recalculated with the updated human GS (6.15 pg), it reduced to 30.54 pg, closely aligning with results from different laboratories and GS measurement techniques (Bennett and Smith, 1976; Doležel et al., 1992, 1998).

The overestimation of GS can lead to inflated resource allocation in genome sequencing projects. For example, the critically endangered *Eidothea hardeniana* was previously estimated to have a GS of 1.81 pg/2C, based on the overestimated GS of ‘Torstag’ pea (2C = 9.49 pg) (Jordan et al., 2015; Pellicer and Leitch, 2020). However, using rice as a reference (2C = 0.788 pg), we re-estimated the GS of *Eidothea* to be 2C = 1.31 pg, which was 37% smaller than the previous estimate (data not shown). The T2T consensus assembly of *Eidothea* (1.26 pg) was slightly smaller than the FCM-based estimate (data not shown). The FCM estimate would have been 2C = 1.23 pg, derived from the phased rice assembly (2C = 0.743 pg). The sum of the haplotype-resolved assemblies for diploid *Eidothea* (2C = 1.22 pg) closely matched the GS estimated using the haplotype-based rice assembly.

Haplotype-resolved genome assemblies of rice demonstrate the progress made at the sequencing level and the suitability of genome assemblies for the recalibration of flow cytometry standards. However, the consideration of extra-chromosomal circular DNA (eccDNA) should not be overlooked, as genome assemblies may represent only the linear chromosomal DNA. In rice, approximately 25,600 eccDNA molecules were identified in various tissues, with nearly 87% found in leaves (Cuzzoni et al., 1990; Zhuang et al., 2024). The length of eccDNA ranged from 74 to 5,000 bp, with the majority between 200 and 400 bp. An average length of 300 bp and approximately 22,180 eccDNA molecules from leaves would account for 6.65 Mbp. Cuzzoni et al. (1990) estimated eccDNA to represent 1% of the total DNA. Similarly, the presence of eccDNA has been reported in *Arabidopsis* and *Nicotiana* (Kinoshita et al., 1985). Given the diverse nature of genome assemblies and eccDNA, genome assemblies may be smaller than FCM estimates due to the exclusion of eccDNA from linear DNA. However, the extent of this difference can only be estimated once eccDNA is identified for each species.

## Data Availability

The flow cytometry experiment files can be accessed directly via the link: dx.doi.org/10.6084/m9.figshare.30304258.

## Supplementary material

Supplementary Figure 1 Comparative analysis of recalibrated estimates (α)- based on previous genome assembly (2C = 0.795 pg) (Sasaki, 2005), recalibrated estimates (β)- based on the complete consensus genome assembly (2C = 0.788 pg), recalibrated estimates (γ)- based on the haplotype resolved genome assembly (2C = 0.743 pg),recalculated estimates, and genome assembly-based estimates.
