# Supplementary figures and images for: Re-calibration of flow cytometry standards for plant genome size estimation
Source: Front Plant Sci. 2025 Oct 13;16:1548766. doi: 10.3389/fpls.2025.1548766 (PMC12555019; doi:10.3389/fpls.2025.1548766)

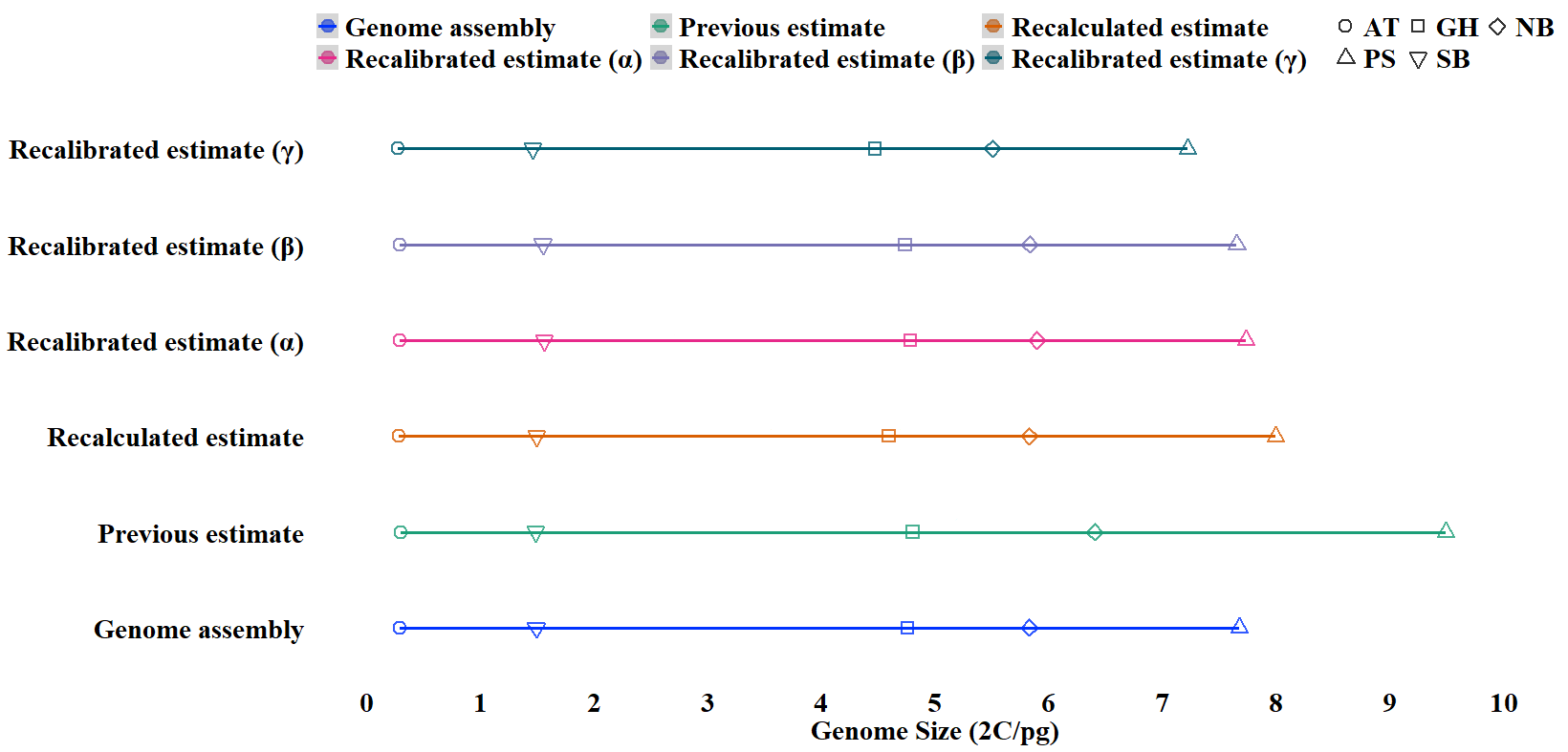

Supplement: Supplementary Figure 1 — Comparative analysis of recalibrated estimates (α)- based on previous genome assembly (2C = 0.795 pg) (Sasaki, 2005), recalibrated estimates (β)- based on the complete consensus genome assembly (2C = 0.788 pg), recalibrated estimates (γ)- based on the haplotype resolved genome assembly (2C = 0.743 pg),recalculated estimates, and genome assembly-based estimates. [file Image1.tiff]
