# Supplementary material for: Re-calibration of flow cytometry standards for plant genome size estimation
Source: Front Plant Sci. 2025 Oct 13;16:1548766. doi: 10.3389/fpls.2025.1548766 (PMC12555019; doi:10.3389/fpls.2025.1548766)
Supplement: Supplementary file 3 [file Table1.docx]

**Supplementary Note 1**

Re-calibration of flow cytometry standards for plant genome size estimation

**Authors**

Abhishek Soni ^1,2*^, Robert J Henry ^1,2*^

^1^Queensland Alliance for Agriculture and Food Innovation, The University of Queensland St Lucia Qld Australia

^2^ARC Centre of Excellence for Plant Success in Nature and Agriculture, The University of Queensland St Lucia Qld Australia

*Correspondence: Abhishek Soni [a.soni@uq.edu.au](mailto:a.soni@uq.edu.au) , [a.soni@mail.com](mailto:a.soni@mail.com) Robert J Henry [robert.henry@uq.edu.au](mailto:robert.henry@uq.edu.au)

**The progress in the human genome sequencing**

The advent of new sequencing technologies has facilitated the resolution of the complex regions of human genome including centromeric satellite repeats, transposable elements, complex structural variation, Chromosome Y and X (including long palindromes, tandem repeats and segmental duplications (Lander et al., 2001;Schneider et al., 2017;Aganezov et al., 2022;Altemose et al., 2022;Morales et al., 2022;Nurk et al., 2022;Vollger et al., 2022;Chao et al., 2023a;Rhie et al., 2023;Li and Durbin, 2024) (Table 2). One of the recently published human genomes, T2T-CHM13v2.0, is now considered gapless and telomere-to-telomere (T2T) (Aganezov et al., 2022;Nurk et al., 2022). With more recent advances and fully sequenced X and Y chromosomes, the current gapless human male genome (44+XY) is around 2C = 6.15 pg (Miga et al., 2020;Nurk et al., 2022;Rhie et al., 2023). Chao et al. (2023b) successfully achieved a gapless genome assembly of a Chinese individual, which closely aligned with the genome size derived from the T2T-CHM13 reference genome assembly. Jarvis et al. (2022) achieved several diploid and haplotype resolved assemblies using a range of sequencing platforms and assembling approaches including CLR reads, CCS reads, ONT reads, ONT Ultra Long reads, Hi-C reads and Bio Nano optimal maps with an assembly size ranging from 5.72 to 6.34 pg in accordance with the expected 2C genome of male 6.16 pg (Table 2). These values were in accordance with the size of the reference genome (T2T-CHM13 v2.0) (Nurk et al., 2022). This high level of congruence demonstrates the precision of genome assembly in representing the human genome. Furthermore, the genome assembly (HG002 Ref.pat) from Jarvis et al. (2022) is ~100% complete when compared to the T2T-CHM13. Likewise, one of the earlier genomes using Sanger sequencing (GRh38.p13) was also 99.53% of the T2T-CHM13 genome (Jarvis et al., 2022). Assembly completeness of the T2T-CHM13 was assessed using k-mer statistics and mapping-based statistics (Nurk et al., 2022). A comprehensive analysis revealed that the T2T-CHM13 assembly maintains uniform coverage across the genome, with 99.86% of the sequence within three standard deviations of the mean coverage for HiFi and ONT reads (Nurk et al., 2022). Excluding the ribosomal DNA (rDNA) sequences, this uniformity further improves to 99.99%. Despite this high level of completeness, some regions of the genome remain associated with potential issues due to low coverage, low confidence, or known heterozygous sites due to the limitations of both ONT and HiFi read challenges in dealing with telomeric regions and GC rich regions respectively (Nurk et al., 2022;Li and Durbin, 2024). These potential issues encompass only 0.3% of the total assembly length, equivalent to approximately 9.165 Mbp, a marked improvement from the 8% of problematic regions observed in GRCh38 (Nurk et al., 2022). This ensures that future research and applications can be guided with greater accuracy and confidence, setting a new standard for genome assembly completeness. Both Han1 (2C = 6.12 pg) and T2T.CHM13v.2.0 (2C = 6.15 pg) are the most complete human genome assemblies (Nurk et al., 2022;Chao et al., 2023a). Considering the reference assemblies and potential sequencing errors in T2T.CHM13v2.0, the complete human male genome was assumed to be 6.15 pg/2C (Table 2). This value agrees with the previous speculations (Doležel et al., 2003;Doležel et al., 2007). Based on this value GS estimates for plant species were recalculated (Table S2).

**Table S2:** The genome size of a recently assembled human male (44Autosomes+XY) genome.

| Assembly name | 2C value (pg) | Sequencing platform | Reference |
| --- | --- | --- | --- |
| Han1 | 6.12 | PacBio high-fidelity (HiFi) reads, and Oxford Nanopore Technology (ONT) reads. | (Chao et al., 2023a) |
|  |  |  |  |
| T2T-CHM13 v.2.0 | 6.15ss | PacBio HiFi CCS reads, PacBio CLR reads, Oxford Nanopore Ultra Long Reads, Illumina short reads, 10x Genomics reads, Hi-C Arima Genomics, Bio Nano optical Maps, Single-Cell DNA temple strand sequencing | (Miga et al., 2020;Nurk et al., 2022;Rhie et al., 2023) |
| HG002 Ref.pat | 6.16 | PacBio HiFi, PacBio CLR, ONT Ultra Long 100Kb+, 10x linked reads, Strand-seq, Hi-C linked reads, Illumina, Bio Nano optical maps and Strand-seq) | (Jarvis et al., 2022) |
| GRCH38.p14 | 6.10 | Shotgun sequencing | (Schneider et al., 2017;Morales et al., 2022) |
| GRCH38 | 5.96 | Shotgun sequencing | (Lander et al., 2001) |

**References**

Aganezov, S., Yan, S.M., Soto, D.C., Kirsche, M., Zarate, S., Avdeyev, P., Taylor, D.J., Shafin, K., Shumate, A., and Xiao, C. (2022). A complete reference genome improves analysis of human genetic variation. *Science* 376**,** eabl3533.

Altemose, N., Logsdon, G.A., Bzikadze, A.V., Sidhwani, P., Langley, S.A., Caldas, G.V., Hoyt, S.J., Uralsky, L., Ryabov, F.D., Shew, C.J., Sauria, M.E.G., Borchers, M., Gershman, A., Mikheenko, A., Shepelev, V.A., Dvorkina, T., Kunyavskaya, O., Vollger, M.R., Rhie, A., Mccartney, A.M., Asri, M., Lorig-Roach, R., Shafin, K., Lucas, J.K., Aganezov, S., Olson, D., De Lima, L.G., Potapova, T., Hartley, G.A., Haukness, M., Kerpedjiev, P., Gusev, F., Tigyi, K., Brooks, S., Young, A., Nurk, S., Koren, S., Salama, S.R., Paten, B., Rogaev, E.I., Streets, A., Karpen, G.H., Dernburg, A.F., Sullivan, B.A., Straight, A.F., Wheeler, T.J., Gerton, J.L., Eichler, E.E., Phillippy, A.M., Timp, W., Dennis, M.Y., O’neill, R.J., Zook, J.M., Schatz, M.C., Pevzner, P.A., Diekhans, M., Langley, C.H., Alexandrov, I.A., and Miga, K.H. (2022). Complete genomic and epigenetic maps of human centromeres. *Science* 376**,** eabl4178.

Chao, K.-H., Zimin, A.V., Pertea, M., and Salzberg, S.L. (2023a). The first gapless, reference-quality, fully annotated genome from a Southern Han Chinese individual. *G3 Genes|Genomes|Genetics* 13.

Chao, K.-H., Zimin, A.V., Pertea, M., and Salzberg, S.L. (2023b). The first gapless, reference-quality, fully annotated genome from a Southern Han Chinese individual. *G3: Genes, Genomes, Genetics* 13**,** jkac321.

Doležel, J., Bartoš, J., Voglmayr, H., and Greilhuber, J. (2003). Nuclear DNA content and genome size of trout and human. *Cytometry Part A* 51**,** 127-128.

Doležel, J., Greilhuber, J., and Suda, J. (2007). *Flow cytometry with plant cells: analysis of genes, chromosomes and genomes.* John Wiley & Sons.

Jarvis, E.D., Formenti, G., Rhie, A., Guarracino, A., Yang, C., Wood, J., Tracey, A., Thibaud-Nissen, F., Vollger, M.R., and Porubsky, D. (2022). Semi-automated assembly of high-quality diploid human reference genomes. *Nature* 611**,** 519-531.

Lander, E.S., Linton, L.M., Birren, B., Nusbaum, C., Zody, M.C., Baldwin, J., Devon, K., Dewar, K., Doyle, M., Fitzhugh, W., Funke, R., Gage, D., Harris, K., Heaford, A., Howland, J., Kann, L., Lehoczky, J., Levine, R., Mcewan, P., Mckernan, K., Meldrim, J., Mesirov, J.P., Miranda, C., Morris, W., Naylor, J., Raymond, C., Rosetti, M., Santos, R., Sheridan, A., Sougnez, C., Stange-Thomann, N., Stojanovic, N., Subramanian, A., Wyman, D., Rogers, J., Sulston, J., Ainscough, R., Beck, S., Bentley, D., Burton, J., Clee, C., Carter, N., Coulson, A., Deadman, R., Deloukas, P., Dunham, A., Dunham, I., Durbin, R., French, L., Grafham, D., Gregory, S., Hubbard, T., Humphray, S., Hunt, A., Jones, M., Lloyd, C., Mcmurray, A., Matthews, L., Mercer, S., Milne, S., Mullikin, J.C., Mungall, A., Plumb, R., Ross, M., Shownkeen, R., Sims, S., Waterston, R.H., Wilson, R.K., Hillier, L.W., Mcpherson, J.D., Marra, M.A., Mardis, E.R., Fulton, L.A., Chinwalla, A.T., Pepin, K.H., Gish, W.R., Chissoe, S.L., Wendl, M.C., Delehaunty, K.D., Miner, T.L., Delehaunty, A., Kramer, J.B., Cook, L.L., Fulton, R.S., Johnson, D.L., Minx, P.J., Clifton, S.W., Hawkins, T., Branscomb, E., Predki, P., Richardson, P., Wenning, S., Slezak, T., Doggett, N., Cheng, J.-F., Olsen, A., Lucas, S., Elkin, C., Uberbacher, E., Frazier, M., et al. (2001). Initial sequencing and analysis of the human genome. *Nature* 409**,** 860-921.

Li, H., and Durbin, R. (2024). Genome assembly in the telomere-to-telomere era. *Nature Reviews Genetics*.

Miga, K.H., Koren, S., Rhie, A., Vollger, M.R., Gershman, A., Bzikadze, A., Brooks, S., Howe, E., Porubsky, D., Logsdon, G.A., Schneider, V.A., Potapova, T., Wood, J., Chow, W., Armstrong, J., Fredrickson, J., Pak, E., Tigyi, K., Kremitzki, M., Markovic, C., Maduro, V., Dutra, A., Bouffard, G.G., Chang, A.M., Hansen, N.F., Wilfert, A.B., Thibaud-Nissen, F., Schmitt, A.D., Belton, J.-M., Selvaraj, S., Dennis, M.Y., Soto, D.C., Sahasrabudhe, R., Kaya, G., Quick, J., Loman, N.J., Holmes, N., Loose, M., Surti, U., Risques, R.A., Graves Lindsay, T.A., Fulton, R., Hall, I., Paten, B., Howe, K., Timp, W., Young, A., Mullikin, J.C., Pevzner, P.A., Gerton, J.L., Sullivan, B.A., Eichler, E.E., and Phillippy, A.M. (2020). Telomere-to-telomere assembly of a complete human X chromosome. *Nature* 585**,** 79-84.

Morales, J., Pujar, S., Loveland, J.E., Astashyn, A., Bennett, R., Berry, A., Cox, E., Davidson, C., Ermolaeva, O., Farrell, C.M., Fatima, R., Gil, L., Goldfarb, T., Gonzalez, J.M., Haddad, D., Hardy, M., Hunt, T., Jackson, J., Joardar, V.S., Kay, M., Kodali, V.K., Mcgarvey, K.M., Mcmahon, A., Mudge, J.M., Murphy, D.N., Murphy, M.R., Rajput, B., Rangwala, S.H., Riddick, L.D., Thibaud-Nissen, F., Threadgold, G., Vatsan, A.R., Wallin, C., Webb, D., Flicek, P., Birney, E., Pruitt, K.D., Frankish, A., Cunningham, F., and Murphy, T.D. (2022). A joint NCBI and EMBL-EBI transcript set for clinical genomics and research. *Nature* 604**,** 310-315.

Nurk, S., Koren, S., Rhie, A., Rautiainen, M., Bzikadze, A.V., Mikheenko, A., Vollger, M.R., Altemose, N., Uralsky, L., Gershman, A., Aganezov, S., Hoyt, S.J., Diekhans, M., Logsdon, G.A., Alonge, M., Antonarakis, S.E., Borchers, M., Bouffard, G.G., Brooks, S.Y., Caldas, G.V., Chen, N.-C., Cheng, H., Chin, C.-S., Chow, W., De Lima, L.G., Dishuck, P.C., Durbin, R., Dvorkina, T., Fiddes, I.T., Formenti, G., Fulton, R.S., Fungtammasan, A., Garrison, E., Grady, P.G.S., Graves-Lindsay, T.A., Hall, I.M., Hansen, N.F., Hartley, G.A., Haukness, M., Howe, K., Hunkapiller, M.W., Jain, C., Jain, M., Jarvis, E.D., Kerpedjiev, P., Kirsche, M., Kolmogorov, M., Korlach, J., Kremitzki, M., Li, H., Maduro, V.V., Marschall, T., Mccartney, A.M., Mcdaniel, J., Miller, D.E., Mullikin, J.C., Myers, E.W., Olson, N.D., Paten, B., Peluso, P., Pevzner, P.A., Porubsky, D., Potapova, T., Rogaev, E.I., Rosenfeld, J.A., Salzberg, S.L., Schneider, V.A., Sedlazeck, F.J., Shafin, K., Shew, C.J., Shumate, A., Sims, Y., Smit, A.F.A., Soto, D.C., Sović, I., Storer, J.M., Streets, A., Sullivan, B.A., Thibaud-Nissen, F., Torrance, J., Wagner, J., Walenz, B.P., Wenger, A., Wood, J.M.D., Xiao, C., Yan, S.M., Young, A.C., Zarate, S., Surti, U., Mccoy, R.C., Dennis, M.Y., Alexandrov, I.A., Gerton, J.L., O’neill, R.J., Timp, W., Zook, J.M., Schatz, M.C., Eichler, E.E., Miga, K.H., and Phillippy, A.M. (2022). The complete sequence of a human genome. *Science* 376**,** 44-53.

Rhie, A., Nurk, S., Cechova, M., Hoyt, S.J., Taylor, D.J., Altemose, N., Hook, P.W., Koren, S., Rautiainen, M., Alexandrov, I.A., Allen, J., Asri, M., Bzikadze, A.V., Chen, N.-C., Chin, C.-S., Diekhans, M., Flicek, P., Formenti, G., Fungtammasan, A., Garcia Giron, C., Garrison, E., Gershman, A., Gerton, J.L., Grady, P.G.S., Guarracino, A., Haggerty, L., Halabian, R., Hansen, N.F., Harris, R., Hartley, G.A., Harvey, W.T., Haukness, M., Heinz, J., Hourlier, T., Hubley, R.M., Hunt, S.E., Hwang, S., Jain, M., Kesharwani, R.K., Lewis, A.P., Li, H., Logsdon, G.A., Lucas, J.K., Makalowski, W., Markovic, C., Martin, F.J., Mc Cartney, A.M., Mccoy, R.C., Mcdaniel, J., Mcnulty, B.M., Medvedev, P., Mikheenko, A., Munson, K.M., Murphy, T.D., Olsen, H.E., Olson, N.D., Paulin, L.F., Porubsky, D., Potapova, T., Ryabov, F., Salzberg, S.L., Sauria, M.E.G., Sedlazeck, F.J., Shafin, K., Shepelev, V.A., Shumate, A., Storer, J.M., Surapaneni, L., Taravella Oill, A.M., Thibaud-Nissen, F., Timp, W., Tomaszkiewicz, M., Vollger, M.R., Walenz, B.P., Watwood, A.C., Weissensteiner, M.H., Wenger, A.M., Wilson, M.A., Zarate, S., Zhu, Y., Zook, J.M., Eichler, E.E., O’neill, R.J., Schatz, M.C., Miga, K.H., Makova, K.D., and Phillippy, A.M. (2023). The complete sequence of a human Y chromosome. *Nature* 621**,** 344-354.

Schneider, V.A., Graves-Lindsay, T., Howe, K., Bouk, N., Chen, H.-C., Kitts, P.A., Murphy, T.D., Pruitt, K.D., Thibaud-Nissen, F., and Albracht, D. (2017). Evaluation of GRCh38 and de novo haploid genome assemblies demonstrates the enduring quality of the reference assembly. *Genome research* 27**,** 849-864.

Vollger, M.R., Guitart, X., Dishuck, P.C., Mercuri, L., Harvey, W.T., Gershman, A., Diekhans, M., Sulovari, A., Munson, K.M., Lewis, A.P., Hoekzema, K., Porubsky, D., Li, R., Nurk, S., Koren, S., Miga, K.H., Phillippy, A.M., Timp, W., Ventura, M., and Eichler, E.E. (2022). Segmental duplications and their variation in a complete human genome. *Science* 376**,** eabj6965.
